# Multimodal AI-based systems in major depressive disorder: a review of clinical and translational applications

**DOI:** 10.3389/fdgth.2026.1812241

**Published:** 2026-04-17

**Authors:** Claudio Crema, Silvia De Francesco, Cesare Michele Baronio, Alberto Boccali, Claudio Demaria, Giovanni Battista Tura, Damiano Archetti, Alberto Redolfi

**Affiliations:** 1 Laboratory of Neuroinformatics, IRCCS Istituto Centro San Giovanni di Dio Fatebenefratelli, Brescia, Italy; 2 Psychiatry Unit, IRCCS Istituto Centro San Giovanni di Dio Fatebenefratelli, Brescia, Italy

**Keywords:** artificial intelligence, clinical, major depressive disorder, multimodal, psychiatry, translational

## Abstract

Major Depressive Disorder (MDD) is one of the most prevalent and disabling psychiatric conditions worldwide, involving alterations in mood regulation, cognitive function, sleep, and physiological systems. Traditional diagnostic approaches often rely on time-consuming interviews and questionnaires, which are largely based on subjective clinical judgment, and may contribute to misdiagnosis or suboptimal treatment selection. Artificial Intelligence (AI) approaches for MDD detection and monitoring have been studied using various data sources, including clinical data, Magnetic Resonance Imaging (MRI), speech features, and genetics. In this review, we collected evidence on multimodal AI-based methods for MDD-related outcomes, focusing on discriminative and predictive performance, validation practices, and feasibility in clinical settings. A search of four databases (PubMed, Web of Science, Scopus, and Embase) was performed, including 40 original studies published after 2015 divided into two main categories: clinical and translational approaches. Our analysis showed that MRI-based biomarkers frequently provide the best performance, but their high cost and time-consuming acquisition limit scalability; simpler measures (audio-visual, clinical, wearable/smartphone digital biomarkers) may offer a better balance between performance and implementability. Reported accuracies are typically between 65%–85%, however a systematic lack of external validation may imply overfitting, highlighting the need for prospective multi-site validation and stratified analyses before clinical translation. Although the landscape is complex, this review suggests that multimodal AI approaches could help clinicians optimize their clinical practices, support decision-making, and monitor patients, thereby improving the quality of healthcare services.

## Introduction

Major Depressive Disorder (MDD) is among the most prevalent and disabling psychiatric conditions worldwide, affecting approximately 3.2% of the world's population in 2021, and representing a leading contributor to global disease burden (Zhang et al., 2025) (1). MDD is highly heterogeneous in symptoms, with marked variability in illness course and treatment response, complicating diagnosis and therapeutic planning (Nemesure et al., 2024) (2), with only about 46% of patients achieving full remission (Dawson et al., 2004) (3). For many, the disorder follows a chronic and recurrent trajectory, characterized by delayed response, repeated treatment trials, and frequent relapse. Current diagnostic and monitoring approaches rely on clinician-conducted interviews and self-report questionnaires, which are time-consuming and based on subjective clinical judgment, contributing to misdiagnosis, delayed intervention, and suboptimal treatment selection [Choi et al., 2020 (4); van Bronswijk et al., 2021 (5)]. Moreover, these methods capture only episodic snapshots of symptomatology, making them poorly suited for continuous monitoring. These limitations underscore the need for objective and biologically informed innovative methods to improve diagnosis and predict clinical outcomes and treatment response at the individual patient level. Artificial Intelligence (AI), and Machine Learning (ML) and Deep Learning (DL) in particular, offer capabilities of identifying complex, non-linear patterns from high-dimensional data. Recent applications [e.g., Abd-Alrazaq et al., 2023 (6),; Lim et al., 2024 (7),; and Kambeitz et al., 2017 (8)] suggest that AI-driven approaches can capture biological variations in MDD not identifiable by traditional methods, offering the potential to move from symptom-checklists-based diagnosis towards biologically grounded precision psychiatry that integrates diverse biomarkers [Comai et al., 2025 (9)]. This integrative approach reflects different schools of thought regarding the optimal sources through which MDD can be characterized: (a) neuroimaging-based biomarkers derived from structural and functional Magnetic Resonance Imaging (MRI) and Diffusion Tensor Imaging (DTI); (b) biomarkers extracted from digital phenotyping via smartphones and wearables, such as audio-visual features and Heart Rate Variability (HRV); (c) clinical and demographic variables; and (d) biological biomarkers including blood samples, genetic variants, and inflammatory markers. The rationale for integrating multimodal biomarkers stems from the complex MDD phenomenology, causing dysfunction across multiple biological systems that cannot be captured with single-modality assessments [Chen et al., 2025 (10)]. While significant advances have been achieved, the use of AI methods could suffer from various issues, such as risk of bias due to small sample sizes and high dimensional data, and risk of overfit due to limited external validation, undermining generalizability across diverse populations and settings. Finally, AI systems in the psychiatric domain, considered a high-risk area for treating vulnerable individuals, must ensure full regulatory compliance, with already deployed systems updated to meet the upcoming requirements of the EU Artificial Intelligence Act (AI-Act) (11).

This review offers a comprehensive, state-of-the-art assessment of the application of multimodal AI tools in MDD research, highlighting their potential to improve diagnosis, treatment selection, and prognosis. It also identifies key future directions, including: (1) characterizing the most promising multimodal AI methods for improving MDD diagnosis and outcome prediction; (2) assessing the quality, generalizability, and clinical utility of current ML and DL models to identify remaining gaps in the literature; and (3) defining operational guidelines and recommendations for the next generation of AI approaches within MDD spectrum.

## Methods

**Figure 1:**
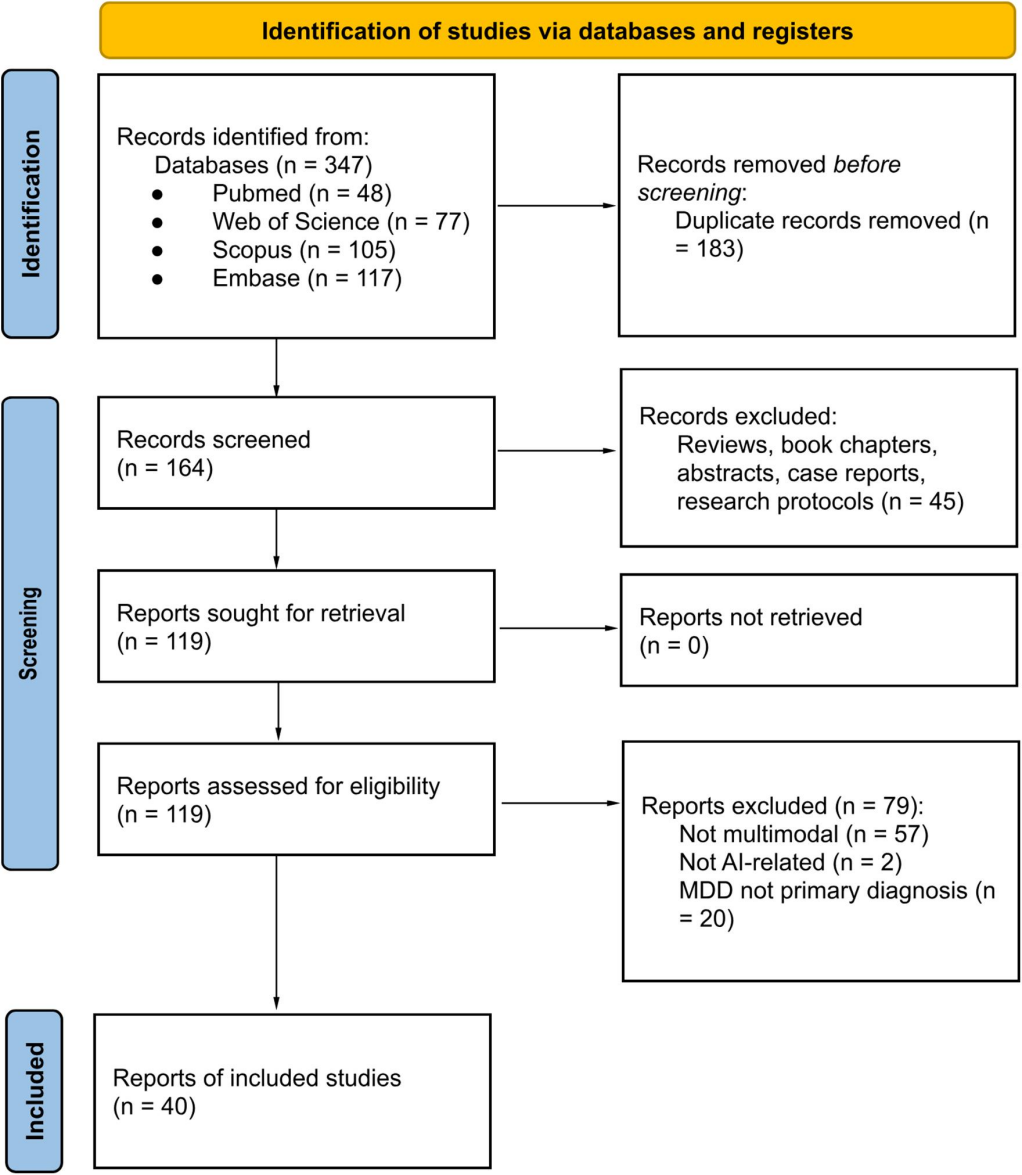
PRISMA chart.

We followed a state-of-the-art narrative review with a systematic search design. The search was conducted on four different repositories (PubMed, Web of Science, Scopus, and Embase) to identify all relevant studies on multimodal AI approaches applied to MDD. The queries included key terms such as: “major depressive disorder”, “artificial intelligence”, “neuroimaging”, “acoustic”, “genetic”, “prediction”, and “classification”. The goal was to retrieve studies on MDD where AI or ML approaches were used in combination with multimodal biomarkers, including neuroimaging, speech and language features, biological markers and genetic data, to predict, classify, or assess depression severity. The content of the queries was the same for every database, only the syntax was adapted to each source. In line with Preferred Reporting Items for Systematic Reviews and Meta-Analyses (PRISMA) recommendations, the full versions of the search queries are described in the Supplementary Material. They were executed in November 2025, searching for papers published since 2015. The search yielded a total of 48 articles from PubMed, 77 from Web of Science, 105 from Scopus, and 117 from Embase, for a total of 347 initial records, reduced to 164 after deduplication. All studies identified in the search were assessed by CC, a neuroscientist with five years of experience in AI applied to clinical contexts. Each study was discussed with the co-authors, and disagreements were resolved by majority vote. Studies were excluded if they were not published in journals ranked in the 2025 Journal Citation Report science edition. Moreover reviews, book chapters, abstracts, non-English articles, case reports, and research protocols were removed. This process resulted in 119 candidate studies that were assessed to understand their relevance to the topic. We defined multimodal AI models as those that combined at least two distinct data domains (e.g., different types of MRI, clinical + MRI, MRI + genomics, speech + HRV) within a single model. Some of the retrieved articles were deemed as out-of-scope: (i) if they were not multimodal, (ii) if not directly related to AI applied to MDD (e.g., some were merely descriptions of datasets, study concepts, or investigated molecular effects of genetic variants without applying AI), (iii) if MDD was not the primary diagnosis of the work (e.g., some focused on schizophrenia or Borderline Personality Disorder, and MDD was a secondary feature). The remaining 40 studies were deemed appropriate and analyzed. Overall, the MDD population considered in this review was represented by adolescents or adults (>15 years old) with a primary diagnosis of MDD defined by Diagnostic and Statistical Manual of Mental Disorders, Fifth Edition, Text Revision (DSM-5-TR) (12). The selection process is summarized in the PRISMA chart, shown in Figure 1.

The following information was extracted from each study:

- Aim of the study;
- Population size;
- Biomarkers used;
- AI methods used (i.e., fusion strategies and type of model);
- Evaluation method;
- Training performance [e.g., accuracy, sensitivity, specificity, and Area Under the Curve (AUC)];
- External/independent test numerosity and performance.

Because of the heterogeneity in the outcome metrics reported across included studies, a direct quantitative comparison of model performance was feasible for a limited number of studies. Where available, AUC was used as the primary metric for classification tasks, as it is widely adopted in the clinical AI literature. For regression tasks (i.e., severity estimation), Mean Absolute Error (MAE) and Root Mean Square Error (RMSE) were reported as primary metrics.

Two main application domains were assessed:

1. Clinical applications (*n* = 30): studies evaluating AI algorithms in clinical contexts for diagnosis or symptom related classification tasks, most of them cross-sectional. This category was further divided into:
  1.1 Diagnostic models (*n* = 19): aimed at distinguishing individuals with MDD from Healthy Controls (HCs). This class was characterized by different multimodal combinations of biomarkers: MRI biomarkers (*n* = 5); MRI + demographic biomarkers (*n* = 3); MRI + biological biomarkers (*n* = 4); Audio-visual biomarkers (*n* = 5); HRV + biological biomarkers (*n* = 2).
  1.2 Differential diagnosis models (*n* = 6): designed to discriminate between depressive subtypes or between MDD and other psychiatric disorders;
  1.3 Symptom severity estimation models (*n* = 5): capable of quantifying the severity of depressive symptoms on a continuous or ordinal scale.
2. Translational applications (*n* = 10): studies applying AI to model biological mechanisms or predict prognostic responses. This category included:
  2.1 Therapy outcome prediction models (*n* = 7): predicting response trajectories to pharmacological or psychotherapeutic interventions;
  2.2 Prognostic outcome prediction models (*n* = 3): forecasting future prognosis including remission, relapse, or psychiatric rehospitalization with a follow-up for prognostic outcomes ranging from 3 to 24 months.

## Results

Several AI concepts are presented in this section. For the sake of the reader, they are briefly explained in the Supplementary material.

### Clinical applications—diagnostic models (MDD vs. controls)

Among studies in this class (Table 1), most used only multimodal MRI data (5 studies), typically combining structural MRI (sMRI), functional MRI (fMRI, predominantly resting-state, rs-fMRI), and/or diffusion tensor imaging (DTI) within a ML pipeline. Common workflows included: modality-specific feature extraction, dimensionality reduction (feature selection or constrained fusion), then classification with Support Vector Machines (SVMs) or DL models. What varied most was when and how modalities were merged (e.g., feature concatenation, network-level fusion, ensemble voting, or self-supervised pretraining on a large dataset followed by fine-tuning on a smaller clinical set). Validation was usually limited to internal cross-validation [including repeated k-fold, nested cross-validation (CV), or Leave-One-Out CV, LOOCV], with almost no truly independent external testing.

**Table 1:** Results of diagnostic models.

Study	AI aims	Population (MDD/HC)	Biomarkers	Method	Evaluation method	Training performance	External/independent test numerosity and performance
Yang (13)	Diagnostic classification (MDD vs. HC)	56/55	fMRI, sMRI	Semi-multimodal early fusion + SVM	5-fold CV	Acc = 84.91 ± 4.19% Sens = 88.60% Spec = 81.29% AUC = 0.91	No
Chen (14)		58/63	fMRI, DTI	Message-passing early fusion + SVM	10-fold CV	Acc = 82.18 ± 10.34% Sens = 77.67 ± 17.36% Spec = 86.33 ± 12.80% AUC = 0.7553 ± 0.1440	No
Pilmeyer (15)		32/31	sMRI, fMRI, DTI	Multi-modal ensemble SVM	Nested LOOCV	Acc = 74.6% [74.2, 74.8%–95% CI] Sens = 78.1% [77.6, 78.5%–95% CI] Spec = 71.0% [70.3, 71.3%–95% CI] AUC = 0.746 [0.741, 0.747%–95% CI]	No
Li (16)		54/62	sMRI, fMRI, DTI	Contrastive learning + hybrid fusion	4-fold CV	Acc = 73.52 ± 2.08% Sens = 74.98 ± 3.10% Spec = 80.00 ± 4.97% AUC = 0.7309	No
Yang (17)		147/52	sMRI, dMRI	Multiple classifiers	10 repeated 5-fold CV	Acc = 74.00 ± 1.32% Sens = NR Spec = NR AUC = 0.74 ± 0.02	Yes (83 MDD, 25 HC) Acc = 75% [65.75, 82.83%–95% CI] Sens = 87.95% [78.96, 94.07%–95% CI] Spec = 32.00% [14.95, 53.5%–95% CI] AUC = 0.6733 [0.5508, 0.7957%–95% CI]
Liu [18]		1113/984	rs-fMRI, sMRI, clinical	Local-to-global multimodal hybrid fusion graph NN	10-fold CV, LOOCV	Acc = 78.85 ± 5.50% Sens = NR Spec = NR AUC = 0.8064 ± 0.0574	Yes Dataset 1 (196 MDD, 177 HC) Acc = 69.97% AUC = 0.7291 Dataset 2 (21 MDD, 21 HC) Acc = 69.05% AUC = 0.7030
Dai [19]		232/393	rs-fMRI, sMRI	mCCA-jICA early fusion + LightGBM	5-fold CV, LOSO	Acc = 99.84 ± 0.12% Sens = 99.77 ± 0.15% Spec = 99.74 ± 0.18% AUC = 0.99995	No
Liu (20)		31/32	DTI, rs-fMRI	Feature selection (t-test filter) + SVM	LOOCV	Acc = 82.26% Sens = 84% Spec = 84% AUC = 0.85	No
Shimizu (21)		91/95	sMRI, rs-fMRI, BDNF Methylation	MCV ensemble of probabilistic learners	10-fold CV	Acc = 80 ± 3% Sens = 73 ± 7% Spec = 87 ± 3% AUC = NR	No
Liang (22)		70/71	dMRI, rs-FMRI, sMRI, miRNA	mCCAR + jICA early fusion + SVM	5 repeated 5-fold CV	Acc = 92.05% Sens = 98% Spec = 86% AUC = 0.96 [0.92, 0.99%–95% CI]	No
Liu (23)		89/61	ASL, rs-fMRI, CTQ scores	Feature integration + SVM	LOOCV with permutation testing	Acc = 70.7% Sens = 73.0% Spec = 65.6% AUC = 0.724	No
Chen (24)		160/119	sMRI, rs-fMRI, AHBA	Feature selection + Kronecker Product early fusion + SVM	100 repeated 10-fold CV	Acc = 83% [80, 97%–95% CI] Sens = 84% [67, 94%–95% CI] Spec = 87% [69, 96%–95% CI] AUC = 0.92 [0.80, 0.97%–95% CI]	Yes, performance NR
Zhang (25)		219 total	Audio, visual, text	Multi-DDAE + Fisher Vector + early fusion + Multitask DNN	10-fold CV	Acc = 85.7% Sens = NR Spec = NR AUC = NR	No
Zhang (26)		18/38	Audio, text	Coarse + fine-grained attention early fusion	3-fold CV	Acc = 73.10% Sens = NR Spec = 55.56% AUC = NR	No
Mamidisetti (27)		82/137	Audio, visual, text	Correlation-based attention weighting + feature weighting + classifier	Train-test split	Acc = 87.3% Sens = 76.9% Spec = NR AUC = NR	Yes (DAIC) Acc = 79.1% Sens = 73.9%
Flores (28)		NR	Audio, visual	Transformers + Explicit alignment + Attention late fusion	Stratified test	Acc = 64% Sens = NR Spec = NR AUC = NR	No
Muzammel (30)		182 total	Audio, visual, text	Model-level late fusion with LSTM	LOOCV	Acc = 77.16% Sens = NR Spec = NR AUC = 0.6575	No
Kim (32)		25/25	Serum proteomics, HRV	Feature selection SVM + multimodal early fusion	1,000 repeated 5-fold CV	Acc = 80.1% Sens = 70.2% Spec = 89.9% AUC = 0.8160 [min = 0.7200, max = 0.8608]	No
Jiang (33)		38/18	Audio, visual, text, rPPG	Early fusion + GBDT/late fusion (majority vote)	100 repeated 5-fold CV	Acc = 76 ± 2% Sens = NR Spec = NR AUC = 0.77 ± 0.01	No

Details of the studies related to binary diagnostic classification. The column “Training performance” contains the best reported results, with standard deviations and confidence intervals, where available. List of acronyms: Acc, accuracy; AHBA, allen human brain atlas; ASL, arterial spin labeling; AUC, area under the curve; BDNF, brain-derived neurotrophic factor; CI, confidence interval; CTQ, childhood trauma questionnaire; DTI, diffusion tensor imaging; DNN, deep neural network; dMRI, diffusion magnetic resonance imaging; fMRI, functional magnetic resonance imaging; GBDT, gradient-boosted decision trees; GNN, graph neural network; HC, healthy controls; HRV, heart rate variability; jICA, joint independent component analysis; LightGBM, light gradient boosting machine; LSTM, long short-term memory; mCCA, multiset canonical correlation analysis; mCCA-JICA, multiset canonical correlation analysis + joint independent component analysis; MCV, maximum credibility voting; MDD, major depressive disorder; miRNA, microRNA; NR, not reported; rPPG, remote photoplethysmography; Res-RNN, residual recurrent neural network; RNN, recurrent neural network; rs-fMRI, resting-state functional magnetic resonance imaging; Sens, sensitivity; sMRI, structural magnetic resonance imaging; Spec, specificity; SVM, support vector machine.

In the case of MRI plus demographics, the studies extended imaging models by adding basic participant variables. Approaches included: (i) a graph Neural Network (NN) that integrated rs-fMRI, sMRI, and demographics (18); (ii) a feature-fusion strategy that first compressed rs-fMRI and sMRI into shared components and then applied a downstream classifier (19); (iii) a DTI connectomics pipeline that derived graph metrics and evaluated an SVM with LOOCV (20). These studies often accounted for multi-site acquisition effects using harmonization (e.g., ComBat) and site-aware validation schemes [e.g., leave-one-site-out, (LOSO)], but only the one by Liu et al. (2024) (18) reported an independent test set.

MRI plus biological biomarkers (4 studies) combined neuroimaging with molecular or biological measures (e.g., DNA methylation, exosomal microRNA) or clinically relevant exposures. The key difference here is the fusion level: some combined modalities at the decision level (train separate models per modality, then aggregate their outputs), while others fused features first (learn shared multimodal components), then performed classification. Most relied on internal resampling for evaluation (repeated k-fold, nested CV, or LOOCV). The study by Chen et al. (2025) (24) validated their model on two independent cohorts by integrating the Allen Human Brain Map (AHBA) with MRI data.

Audio-visual signals were the second most common biomarker type (5 studies) and were used for binary diagnostic classification from clinical interviews. These studies typically combined three information streams: (i) speech acoustics (e.g., pitch and voice-quality measures), (ii) visual behavior (e.g., gaze, head pose, facial activity), and (iii) language features derived from transcripts (e.g., word-use patterns or sentiment/affect markers). Studies differed in where integration happens: some fused features early (combine audio-visual and text features first, then train one model); others used attention mechanisms to merge modality-specific representations inside the model (29); Muzammel et al. (2021) (30) compared multiple architectures and fusion strategies on a benchmark dataset [Distress Analysis Interview Corpus—Wizard of Oz (DAIC-WOZ) (31)]. Validation procedures were heterogeneous and often limited to internal CV or dataset-specific splits. Only Mamidisetti and Reddy (2024) (27) evaluated on the DAIC independent dataset, which is the strongest design for assessing generalizability.

A few studies focused on non-imaging physiological signals, with HRV as primary biomarkers for MDD diagnosis. Kim et al. (2017) (32) combined electrocardiogram (ECG)-derived HRV with serum proteomic markers and trained an SVM with recursive feature elimination and repeated 5-fold CV. Jiang et al. (2024) (33) extracted remote photoplethysmography (rPPG) signals from tele-video interviews to derive HRV features and tested several binary classification tasks with 100 repeated 5-fold CV. These studies yielded modest results, but again without external testing.

### Clinical applications—differential diagnosis

Seven studies addressed differential diagnosis (Table 2). Most (3 studies) focused on distinguishing MDD from Bipolar Depression (BD), while the others targeted different distinctions: MDD vs. Generalized Anxiety Disorder (GAD), MDD vs. Treatment-Resistant Depression (TRD), MDD vs. schizophrenia in a multi-class setting, and one study that split MDD into cognitive subtypes.

**Table 2:** Results of differential diagnosis.

Study	AI aims	Population	Biomarkers	Method	Evaluation method	Training performance	External/independent test numerosity and performance
Vai (34)	Differential diagnosis (MDD vs. BD)	MDD = 74 BD = 74	sMRI, DTI	MKL + SVM	Nested 10-fold CV	Acc = 73.65% Sens = 74.32% Spec = 72.97% AUC = 0.79	No
Lee (35)		MDD = 147 BD = 78	sMRI, WES	Feature extraction + genomic model + late fusion + softmax	10-fold CV	Acc = 72.43 ± 3.16% Sens = 85.12 ± 2.64% Spec = 69.66 ± 7.40% AUC = 0.8912 ± 0.0163	No
Chen (38)		MDD = 114 BD = 117	sMRI, rsMRI	Feature selection + SVM/NN	10 repeated 5-fold CV	Acc = 88.24% Sens = 76.92% Spec = 95.24% AUC = NR	No
Hilbert (39)	Differential diagnosis (MDD vs. GAD)	MDD = 14 GAD = 19	Clinical, hormonal, sMRI	Multimodal late fusion + SVM	Nested LOOCV, permutation testing	Acc = 67.46% Sens = 77.78% Spec = 57.14% AUC = NR	No
Colombo (40)	Differential diagnosis (MDD vs. TRD)	MDD = 71 TRD = 31	sMRI, DTI	Unsupervised multimodal clustering	10 repeated 2-fold CV	Acc = 67% Sens = NR Spec = NR AUC = NR	No
Richter (41)	Differential diagnosis (MDD vs. Schizo)	MDD = 83 Schizo = 41	Audio, visual	Feature-level multimodal early fusion + MLP	10 repeated 10-fold CV	Multiclass performance MDD: Sens = 64%, Spec = 88% Schizo: Sens = 72%, Spec = 91%	No

Details of the studies related to differential diagnosis. The column “AI aims” reports the diagnostic classes investigated by every study, grouped if more studies were related to the same diagnostic class. The column “Training performance” contains the best reported results, with standard deviations and confidence intervals, where available. List of acronyms: Acc, accuracy; AUC, area under the curve; BD, bipolar disorder; CV, cross-validation; DTI, diffusion tensor Imaging; LOOCV, leave-one-out cross-validation; MDD, major depressive disorder; MKL, multiple Kernel learning; MLP, multilayer perceptron; NN, neural network; NR, not reported; rsMRI, resting-state magnetic resonance imaging; Schizo, schizophrenia; Sens, sensitivity; sMRI, structural magnetic resonance imaging; Spec, specificity; SVM, support vector machine; TRD, treatment-resistant depression; WES, whole-exome sequencing.

Overall, the “MDD vs. BD” studies followed a similar workflow: they combined information from more than one source, often multiple MRI sequences, or neuroimaging plus omics [whole-exome sequencing (WES), single-nucleotide polymorphism (SNP), polygenic risk scores (PRS)], inflammation markers, or cognitive measures, then trained a supervised classifier, e.g., Extreme Gradient Boosting (XGBoost) (36) or Light Gradient-Boosting Machine (LightGBM) (37). Studies differed in how data modalities were fused and the evaluation strategy employed [e.g., repeated random splits, k-fold (nested) CV]. However, most results relied only on internal resampling rather than testing on a fully independent external cohort.

The studies that differentiated between MDD and other mood disorders shared a multimodal rationale, but their methodology differed fundamentally in objective and validation logic. Hilbert et al. (2017) (39) combined clinical questionnaires, salivary cortisol, and MRI-derived measures to separate MDD from GAD using a multimodal ensemble approach and nested validation. Colombo et al. (2024) (40) approached MDD vs. TRD by first using MRI for unsupervised clustering, then testing whether those cluster labels could be predicted in a supervised setting, with scanner harmonization (ComBat) included. Richter et al. (2024) (41) used remotely collected audio-visual behavioral markers for four-class classification including MDD and schizophrenia. None of these studies reported tests on independent datasets, limiting the generalizability of the results.

### Clinical applications—symptom severity estimation

The aim of these studies was to predict depression severity, measured with standardized rating instruments such as: Hamilton Depression Rating Scale (HAMD), Beck Depression Inventory-Second Edition (BDI-II), Patient Health Questionnaire (PHQ)-8/9, or Montgomery-Åsberg Depression Rating Scale (MADRS). Audio-visual interview studies analyzed with supervised ML (e.g., Random Forest, RF) found that combining data modalities predicts PHQ severity better than any single modality (Julia et al., 2025 (43)), with minimal errors. Results from multimodal MRI studies [Maglanoc et al., 2019 (45)] were more modest and less consistent, especially for cross-sectional symptom-load prediction; for instance, Wade et al. (2021) (44) predicted symptom reduction after serial ketamine infusions.

Data fusion was implemented in the pipelines in heterogeneous manners. Rohanian et al. (2019) (42) implemented word-level temporal alignment and multimodal sequence fusion on interview data, whereas Julia et al. (2025) (43) performed multimodal integration at the level of entire session-derived features (acoustic, linguistic, facial) within a longitudinal mixed-effects modeling framework. Zhang et al. (2024) (46) emphasized a hybrid fusion design using an attention-based decision module, implemented via encoders plus Bidirectional Long Short-Term Memory (BiLSTM) for temporal modeling.

Nearly all papers used supervised regression and reported MAE and RMSE (sometimes also explained variance), but evaluation was mostly based on internal resampling or CV rather than external test cohorts, which limited claims about generalizability. Across the studies reported in Table 3, these models showed moderate accuracy for severity estimation. A more quantitative performance analysis was not feasible due to substantial methodological heterogeneity across studies, including divergence in target severity scales (PHQ-8, HAMD-17, BDI-II), error metrics (MAE, RMSE), and evaluation frameworks (train-test splits vs. repeated nested CV). Notably, RMSE is systematically larger than MAE for the same model, as it disproportionately penalizes larger prediction errors; thus, these metrics are not directly interchangeable across studies.

**Table 3:** Results of symptom severity estimation.

Study	AI aims	Population	Biomarkers	Method	Evaluation method	Training performance	External/independent test numerosity and performance
Rohanian (42)	PHQ-8 severity prediction	DAIC-WOZ corpus	Audio, visual, lexical	Word-level multimodal early fusion + LSTM	DAIC-WOZ split	MAE = 3.61 RMSE = 4.99	No
Julia (43)		954 total participants	Audio, visual, lexical	Multimodal mixed-effects approach	5-fold nested CV	MAE = 2.43 ± 0.11 MSE = 10.40 ± 0.95	No
Wade (44)	HAMD-17 + HAMD-6 severity prediction	MDD = 60 HC = 19	rs-fMRI, sMRI, dMRI	RF regressor	10 repeated 10-fold CV	HAMD-17 prediction = 19%, HAMD-6 prediction = 27%,	No
Maglanoc (45)	BDI-II severity prediction	MDD = 170 BD = 71	sMRI, DTI, rs-fMRI	LICA early fusion (47)	100 repeated 10-fold CV	Poor performance for symptom prediction	No
Zhang (46)	Severity score regression	AVEC2013 (48), AVEC2014 (49)	Multimodal (Behavioral/Audio-visual implied)	Hybrid fusion + BiLSTM	Train-test split	AVEC2013: MAE = 6.48 RMSE = 8.91 AVEC2014: MAE = 7.01 RMSE = 9.38	No

Details of the studies related to symptom severity estimation. The column “AI aims” reports the standardized rating instruments investigated by every study, grouped if more studies were related to the same instrument. The column “Training performance” contains the best reported results, with standard deviations and confidence intervals, where available. List of acronyms: AI, artificial intelligence; AVEC, audio-visual emotion challenge (dataset/workshop); BD, bipolar disorder; BDI-II, beck depression inventory-II; BiLSTM, bidirectional long short-term memory; CV, cross-validation; DAIC-WOZ, distress analysis interview corpus—wizard of Oz; dMRI, diffusion magnetic resonance imaging; DTI, diffusion tensor imaging; HAMD-6, Hamilton depression rating scale (6-item version); HAMD-17, Hamilton depression rating scale (17-item version); HC, healthy controls; LICA, linked independent component analysis; LSTM, long short-term memory; MAE, mean absolute error; MDD, major depressive disorder; MSE, mean squared error; PHQ-8, patient health questionnaire-8; RF, random forest; RMSE, root mean squared error; rs-fMRI, resting-state functional magnetic resonance imaging; sMRI, structural magnetic resonance imaging.

### Translational modeling—therapy outcomes prediction

Of the seven studies examined treatment outcome prediction (response/remission or symptom change, see Table 4), six of them were related to pharmacotherapy, typically selective serotonin reuptake inhibitor (SSRI) trials. Most models were based on ML and used baseline or early-treatment predictors, mainly neuroimaging features often combined with clinical variables and, in some cases, blood or electroencephalography (EEG) measures, and reported encouraging performance under internal validation.

**Table 4:** Results of therapy outcomes prediction.

Study	AI aims	Population	Biomarkers	Method	Evaluation method	Training performance	External/independent test numerosity and performance
Poirot (50)	Early-treatment sertraline response	MDD = 229, sertraline = 146, placebo = 150	Task-based fMRI, rsMRI, clinical	Feature-level multimodal integration + XGBoost	Nested CV	Acc = 68 ± 10% AUC = 0.73 ± 0.03	No
Sajjadian (51)	Treatment outcome prediction	MDD = 188	Clinical, blood, sMRI, DTI, rsMRI, task-based fMRI	Feature-level multimodal integration + several ML models (elastic net, RF, GBM, SVM, NB)	100 repeated Nested CV	Acc = 66% Sens = 59% Spec = 73% AUC = 0.71	No
Schultz (52)	Treatment outcome prediction (serotonin reuptake inhibitors (*N* = 11), Alpha2-receptor antagonists (*N* = 6), atypical antipsychotics (*N* = 5)	MDD = 21 HC = 20	Task-based fMRI, rs-fMRI	Decision-level early fusion + SVM	LOOCV with permutation testing	Acc = 75.9%, CI = 4.5%	No
Poirot (53)	Sertraline treatment arm outcome prediction	MDD = 262	sMRI, clinical	Primarily unimodal with clinical predictors as additional covariates/features + ML classifiers (SVC, gradient boosting, ResNet)	10-fold CV, LOOCV, permutation testing	Acc = 50.5%	No
Nguyen (54)		MDD = 222, sertraline = 106, placebo = 116, bupropion = 37	Reward-task fMRI, clinical, demographic	Multimodal deep learning with early fusion + NN	Nested CV with permutation testing	Sertraline: R^2^ = 0.48 Response discrimination: AUC = 0.62	No
Jiao (55)	HAMD change prediction	MDD = 296, sertraline = 130, placebo = 135	rs-fMRI, rs-EEG	Representation-level multimodal hybrid fusion + MLP	10-fold CV with permutation testing	R^2^≈0.31 (sertraline) R^2^≈0.28 (placebo)	No
Kravchenko (56)	ICBT outcome prediction	MDD = 1,300, PD = 727, SAD = 641	Clinical, register-based, PRS	Multisource regression (no biomarker fusion)	Explained variance (adjusted R^2^, AIC, RMSE).	Explained variance = 34%	No

Details of the studies related to therapy outcomes prediction. The column “AI aims” reports the goal of each study, grouped if more studies were related to the same instrument. The column “Training performance” contains the best reported results, with standard deviations and confidence intervals, where available. List of acronyms: Acc, accuracy; Accuracy, accuracy; AIC, akaike information criterion; AUC, area under the curve; CI, confidence interval; CV, cross-validation; DTI, diffusion tensor imaging; EEG, electroencephalography; fMRI, functional magnetic resonance imaging; GBM, gradient boosting machine; HAMD, Hamilton depression rating scale; HC, healthy controls; LOOCV, leave-one-out cross-validation; MDD, major depressive disorder; ML, machine learning; MLP, multilayer perceptron; NB, naive Bayes; NN, neural network; PD, panic disorder; PRS, olygenic risk score; R2, coefficient of determination (R²); ResNet, residual network; RF, random forest; RMSE, root mean squared error; rs-EEG, resting-state electroencephalography; rs-fMRI, resting-state functional magnetic resonance imaging; rsMRI, resting-state magnetic resonance imaging; SAD, social anxiety disorder; sMRI, structural magnetic resonance imaging; Sens, sensitivity; Spec, specificity; SVC, support vector classifier; SVM, support vector machine; XGBoost, eXtreme gradient boosting.

Although methodologically different, all the pharmacotherapy papers had a common aim: learn a model that maps pre-treatment biomarkers to later symptom change. Some studies [Poirot et al., 2024 (50), Sajjadian et al., 2023 (51)] tested generalization by training in one trial arm and testing in another (e.g., the sertraline arm vs. placebo, or placebo non-responders subsequently switched to sertraline). Others relied on small-sample CV [including LOOCV, with Schultz et al. (2018) (52) explicitly warning about overfitting], while one large multi-cohort mega-analysis by Poirot et al. (2025) (53) found structural MRI markers offered little or no predictive value beyond chance at the group level. Two Establishing Moderators and Biosignatures of Antidepressant Response for Clinical Care (EMBARC) Randomized Clinical Trial studies by Nguyen et al. (2022) (54) and Jiao et al. (2025) (55) used richer multimodal designs but differed in how they represented imaging (task activation vs. connectivity graphs) and in how they fused modalities (simple concatenation vs. representation-level fusion), while the only psychotherapy study by Kravchenko et al. (2025) (56) focused on Inference-based Cognitive Behavioral Therapy (ICBT) predicted post-treatment severity using clinical variables plus PRS. Notably, none of the studies reported validation on independent external datasets.

### Translational modeling—prognostic outcome prediction

All the prognostic studies aimed at long-term outcome prediction (e.g., remission course or rehospitalization) from baseline multimodal data, and they mainly evaluated performance using internal resampling or a single hold-out split rather than independent cohorts. Two papers by Habets et al. (2023) (57) and Cearns et al. (2019) (58) used baseline clinical variables plus broad biological panels to predict 2-year outcomes such as remission status or rehospitalization. The study by Wallert et al. (2022) (59) predicted remission after ICBT using a mixed feature set of predictors, i.e., demographic, clinical, digital platform behavior, and genetic PRS. Methodologically, these papers differed more in how multimodal integration was operationalized and validated through feature-importance and explainable analyses based on SHapley Additive exPlanations (SHAP) (60) or elastic-net selection. None of the three studies employed independent external testing (see Table 5).

**Table 5:** Results of prognostic outcome prediction.

Study	AI aims	Population	Biomarkers	Method	Evaluation method	Training performance	External/independent test numerosity and performance
Habets (57)	2-years remission prediction	MDD = 804	Clinical, blood proteomics, lipid metabolomics, transcriptomics, PRS	Early fusion + XGBoost + SHAP feature importance analysis	Nested 10-fold CV	AUC = 0.78	No
Wallert (59)		MDD = 894 total	Demographic, clinical, digital-platform behavior, PRS	Early fusion (optional late fusion) + RF	3 repeated 7-fold CV	Acc = 65.6% AUC = 0.687	No
Cearns (58)	2-years rehospitalization prediction	MDD = 380	Clinical, sMRI, serum biomarkers, PRS, cardiovascular, body composition, medication-type predictors	Early fusion + SVM	Nested 10-fold CV with permutation testing	Acc = 63.05% AUC = 0.6774	No

Details of the studies related to prognostic outcome prediction. The column “AI aims” reports the prediction investigated by every study, grouped if more studies were related to the same instrument. The column “Training performance” contains the best reported results, with standard deviations and confidence intervals, where available. List of acronyms: Acc, accuracy; AUC, area under the curve; CV, cross-validation; MDD, major depressive disorder; PRS, polygenic risk score; RF, random forest; sMRI, structural magnetic resonance imaging; SHAP, SHapley additive exPlanations; SVM, support vector machine; XGBoost, eXtreme gradient boosting.

## Discussion

Our review identified multimodal approaches as the dominant methodological framework in AI-based systems; however, their predictive performance must be considered in relation to biomarker accessibility, cost burden, and generalizability constraints, which determine their real-world utility.

### Clinical studies—diagnostic classification

Among the 21 studies addressing binary classification of MDD vs. HC, the highest reported accuracy was achieved by multimodal neuroimaging pipelines integrating sMRI, fMRI, and DTI within fusion frameworks. Most studies implemented classical ML models, with SVM being the most prevalent. SVMs are well-established models that, compared with more recent DL algorithms, have the intrinsic advantage of requiring a much smaller training dataset to be effective, typically requiring some tens or a few hundred samples, while DL models typically require tens of thousands of samples. However, the reported metrics require careful interpretation, as the absence of fully independent external validation could indicate overfitting. A critical observation emerged from studies employing external validation: Yang et al. (2018) (13) demonstrated that multimodal MRI classification accuracy of 75% in the training set dropped to 32% specificity in an independent test cohort, exemplifying how model complexity and feature dimensionality can capitalize on dataset-specific properties rather than generalizable disease signatures. This pattern aligns with recent guidance (61, 62), which emphasizes that internal CV, including nested approaches, does not guarantee generalizability, particularly when acquisition protocols, scanner hardware, or demographic composition differ. The practical implication is that MRI-based biomarkers, despite superior discriminative capacity in controlled settings, face substantial barriers to clinical deployment: MRI scanners require high maintenance costs, lengthy acquisition protocols, and specialized technician staffing, making population-level screening via AI systems economically unfeasible.

On the other hand, interview-derived audio-visual biomarkers, the second most prevalent approach in the reviewed studies, can be acquired with inexpensive and standard audiovisual equipment, such as microphones and cameras available on smartphones or personal computers. However, most of these studies, although benefiting from publicly available datasets (e.g., DAIC-WOZ), lacked external validation. A notable exception is Mamidisetti and Reddy (2024) (27), where independent validation was performed, reporting an accuracy of 79.1% vs. a training-set accuracy of 87.3%, demonstrating performance degradation but maintaining clinically useful discriminative capability. In addition, audio-visual-based studies could benefit from recent improvements in Natural Language Processing (NLP) Large Language Models (LLMs) in particular, which can be used to implement automatic speech-analysis pipelines. This offers a substantial scalability advantage, as audio-visual assessment can be conducted remotely, requires minimal infrastructure, and enables low-cost repeated administration. Although these approaches typically report AUCs below the highest MRI-fusion benchmarks, their cost-effectiveness favors them for population screening and early identification workflows.

Models based on HRV biomarkers demonstrated AUCs in the range of 0.7–0.8, depending on configuration and multimodal fusion strategy. Notably, one study employed rPPG extracted from tele-video interview data, enabling HRV-derived features to be obtained without dedicated sensors. External validation studies of rPPG have established its technical feasibility for remote, contact-free assessment, simplifying the dissemination and adoption of these AI tools. Indeed, HRV-based monitoring offers longitudinal, high-frequency sampling suitable for therapy outcome tracking.

Regardless of the adopted class of biomarkers, when validation with independent datasets is missing, particular caution must be adopted, especially in case of exceptionally good results [e.g., Dai et al. (19) report an extremely high accuracy of 99.84%], as this could be a major sign of overfit.

### Clinical studies—differential diagnosis

The studies addressing differential diagnosis represent a more challenging classification task, as phenotypic boundaries between conditions show considerable overlap, particularly between MDD and BD (63). As in diagnostic models, most studies implemented classical SVM models, although few studies adopted DL algorithms. Multimodal neuroimaging approaches achieved the highest reported AUCs in the 0.8–0.9 range. A critical methodological limitation is the systematic absence of external validation across differential diagnosis studies, which may limit generalizability. An interesting finding emerged from Hilbert et al. (2017) (39): when combining clinical questionnaires, salivary cortisol, and structural MRI in a multimodal ensemble for MDD vs. GAD discrimination, the single-modality cortisol model outperformed the full multimodal model. This demonstrates that multimodal fusion does not always guarantee higher performance and that AI performance are influenced by the features used, sample size, and the complexity of AI architecture, which must be optimized with each problem.

Audio-visual digital biomarkers remain substantially underdeveloped for differential diagnosis. Preliminary evidence from speech and facial kinematics in a four-class differential diagnostic setting (including MDD) showed modest performance, with no external validation reported. These results underscore that differential diagnosis using multimodal digital biomarkers still requires significant methodological advancement and rigorous external validation before clinical implementation.

### Clinical studies—symptom severity estimation

Five studies addressed depression severity prediction using standardized rating instruments (PHQ-8, PHQ-9, HAMD, BDI-II), with the dominant approach employing interview-derived audio-visual features. The most commonly used model in this category is an RF regressor, a well-established ML algorithm that typically achieves good performance with limited data (64). One study used a DL model, but its performance was comparable to the RF-based pipelines. Studies using models with multimodal fusion achieved good predictive accuracy in PHQ-8 prediction, outperforming single-modality ones. When severity modeling is modelled as a longitudinal monitoring task, MRI biomarkers, although potentially reflecting important disease biology, may be impractical due to cost and clinical burden, resulting in insufficient temporal resolution necessary for longitudinal severity monitoring in clinical care. In contrast, audio-visual and speech-derived biomarkers can be sampled repeatedly via smartphone or interview-based platforms, enabling high-frequency longitudinal tracking at minimal marginal cost. Moreover, this class of biomarkers can benefit from NLP/LLM/Generative-AI models, unlocking additional features related to speech content (such as sentiment analysis and lexical frequency patterns) that could assist regressor estimates. In summary, audio-visual and speech-derived multimodal features appear more suitable for severity prediction and longitudinal monitoring than MRI-only approaches, as they offer repeatable acquisition at low cost and enable remote administration. As in previous cases, none of the studies reviewed in this category reported external validation, which limits generalizability and clinical implementation.

### Translational studies—therapy outcome

Among the seven translational studies addressing the prediction of pharmacological or psychotherapeutic outcomes, most used traditional ML models (SVMs), yielding modest performance. However, those applying DL architectures did not perform substantially better. Multimodal MRI ensembles reported the highest performance, achieving AUCs in the 0.6–0.7 range under internal validation for predicting 6-month treatment outcomes, but external validation data were consistently absent from these studies. A critical counterexample was provided by Poirot et al. (2025) (53), who conducted a multi-cohort mega-analysis integrating cortical morphometry and clinical variables across diverse sites, achieving performance at chance level in the overall sample, emphasizing that neuroimaging-only biomarkers lack sufficient generalizability for clinical-scale pharmacotherapy response prediction. Studies employing multimodal baseline and early-treatment neuroimaging with ML reported modest performance metrics likely inflated by limited external validation. Interestingly, AUCs observed in translational tasks are, on average, lower than those reported for diagnostic classification tasks. This could be caused by the prediction problem itself, as therapy outcome prediction must capture dynamic, multi-factorial biological responses to treatment, rather than discriminating disease signatures. Bridging this gap will require longitudinal study designs and larger training samples than those available in the current literature.

### Translational studies—prognostic

Three studies addressed prognosis by predicting long-term outcomes, including disease course, remission status, or risk of rehospitalization using baseline biological and clinical features. The studies used traditional ML models (namely XGBoost, SVM, and RF) and achieved modest to good performance. Across all these prognostic studies, the main limitation was the absence of validation on independent data, precluding confident assessment of generalizability to other clinical populations or treatment settings. Generally, prognostic biomarker prediction requires substantial methodological investment in longitudinal sampling, external validation across diverse clinical populations, and explicit cost-effectiveness analysis before translation of AI prognostic tools into clinical practice.

### Final consideration and recommendations

Across studies, the core challenge appears to be whether high-performance AI systems can be improved or transformed into tools that function effectively in real clinical scenarios. High-performing biomarkers (e.g., imaging, omics) are expensive, time-consuming to process, and difficult to scale, especially in longitudinal frameworks, while simpler or digital signals can support screening and monitoring but may sacrifice peak accuracy. The cost-per-accuracy-gain of collecting each biomarker is an underexplored aspect of the translation of multimodal AI into clinical practice. MRI-based models achieve AUCs in the 0.7–0.9 range for clinical applications, but they require expensive infrastructure and qualified personnel. Speech and audio-visual biomarkers yield lower AUCs, in the 0.6–0.8 range, but they are obtainable at negligible per-patient cost (65). No study to date has formally quantified the incremental accuracy gained per unit cost when adding neuroimaging to scalable digital modalities. Once a sufficient number of studies has accumulated in the literature, simulation-based analyses could be conducted to determine whether there is a performance gain in adopting expensive modalities relative to a marginal improvement of 5%–10% in performance metrics. Future research should explicitly model this trade-off, reporting not only peak performance but cost-weighted performance metrics, to inform evidence-based decisions about modality selection in resource-constrained clinical settings.

An advantage of using audio-visual, speech-derived, and HRV-based biomarkers is the possibility to implement digital phenotyping, defined as the “moment-by-moment assessment of an illness state through digital means” (66). This approach could represent a methodological bridge between episodic clinical assessments and continuous AI-driven care: rather than relying on episodic evaluations, it enables continuous and longitudinal data collection that can feed AI pipelines in real time. In the context of MDD, digital phenotyping could support early detection of symptom worsening and relapse prediction based on longitudinal trajectories of behavioural biomarkers. Future research should implement multimodal AI systems within a digital phenotyping framework, reporting not only cross-sectional diagnostic accuracy but longitudinal predictive validity.

A further point of investigation could be the comparison of early-fusion vs. late-fusion strategies, as the choice of fusion technique represents a potentially important methodological variable. However, among the studies included in the present review, only a limited subset adopted late-fusion strategies, thus making a statistically robust cross-study analysis unfeasible. We acknowledge this as a limitation of the present review.

AI-model selection for each of the five categories described must consider data constraints, and all the challenges described in the present review have motivated operational guidelines and recommendations for the development of the next generation of AI systems, as reported in Box 1.

BOX 1 **Operational guidelines for multimodal AI systems in MDD.**

**Table:** 

1	To define the *intended use* of the AI system under development (i.e.: disease diagnosis vs. differential diagnosis vs. severity monitoring vs. outcome prediction vs. prognosis prediction) stating human-AI interaction (i.e.: human in the loop) as requested by AI-act article 14 (Human oversight: “AI systems must be designed in a way that allows humans to effectively oversee them. The goal of human oversight is to prevent or minimize risks to health, safety, or fundamental rights that may arise from using these systems”) and 13 (Transparency and provision of Information to Deployers: “AI systems must be designed to be transparent, so that those using them can understand and use them correctly”).
2	To define *validation strategy* of the AI system under development (prioritizing truly independent external validation strategies)
3	To treat *multimodal data integration* as a cost/benefit problem: quantify incremental value of each modality compared to unimodal and justify modality burden: MRI/omics vs. scalable digital biomarkers. It is important to report acquisition factors (device, setting, protocols) and test robustness of AI systems to these shifts
4	To define how *missing data* are treated and mitigated (e.g.: dropout, imputation, modality-conditional models)
5	To prevent *leakage errors* [i.e.: all normalization, harmonization (ComBat), feature selection, and tuning must occur in the training-only partitions] and use data leakage safe evaluation designs (i.e.: LOOCV, LOSO, nested CV) to reduce optimistic bias
6	To report *calibration* and decision *relevant metrics* (calibration, clinically meaningful thresholds), not only best AUC/accuracy (especially for small sample)
7	To adopt *reporting checklists* matched to study type (e.g.: STARD-AI for diagnostic accuracy, PROBAST-AI for classification, TRIPOD-AI for prognostic models) to detect hidden biases and promote model transparency

This review highlighted a recurring gap between internal cross-validation and independent performance. Reported results decline on truly independent cohorts due to data leakage, overfitting, and population shift. Critically, independent validation represents the methodological gold standard for performance estimation, as independent test sets (e.g., established repositories like DAIC-WOZ) should be used to estimate the performance degradation that occurs when models are deployed beyond their training distribution. Internal cross-validation is insufficient as a primary evidence base for clinical utility claims. Of utmost importance, next-generation AI tools should treat the external validation process as a primary endpoint, not as a “future work” add-on.

Moreover, other possible sources of bias must be taken into consideration when assessing the real-world applicability of AI-based systems. The majority of the studies selected in this review recruited relatively small samples, often from a single clinical center. This is a selection bias that may inflate model performance and restrict generalizability to broader clinical populations. Furthermore, the heterogeneity of diagnostic instruments (e.g., DSM-IV vs. DSM-5) across studies could introduce label noise that may affect both model training and performance estimation. Finally, the absence of standardised acquisition protocols for behavioural and neuroimaging biomarkers introduces inter-site and inter-device variability that is rarely quantified.

Claims of utility should therefore be supported by realistic performance estimates obtained from independent datasets after all the aforementioned potential sources of bias have been assessed. In light of this, open repositories and benchmark datasets are essential and must be developed in line with open science and FAIR principles to enable further testing and support the maturation of innovative AI tools thereby facilitating their translation from research to clinical practice.

## Conclusions

Multimodal AI-based systems in MDD are progressing from proof-of-concept to potential clinical utility. Low-burden digital and physiological signals are more scalable and cost-effective than imaging or omics, but typically showed modest performance. A key gap remains: AI systems that perform well in controlled, high-dimensional research settings often lack evidence of generalizability beyond their development training cohorts. As a result, support for clinical and translational effectiveness is still limited, with too few external validation studies to demonstrate real-world applicability of AI systems. The next phase should shift the focus from designing better AI systems to generating stronger real-world evidence through multi-site evaluations, standardized data acquisition, and validation strategies that explicitly measure generalizability across populations, sites, and care pathways.
