# Supplementary material for: Multimodal AI-based systems in major depressive disorder: a review of clinical and translational applications
Source: Front Digit Health. 2026 Apr 17;8:1812241. doi: 10.3389/fdgth.2026.1812241 (PMC13133013; doi:10.3389/fdgth.2026.1812241)
Supplement: Supplementary file 1 [file Table1.docx]

Supplementary Material

Claudio Crema^1*^, Silvia De Francesco^1^, Cesare Michele Baronio^1^, Alberto Boccali^1^, Claudio Demaria^1^, Giovanni Battista Tura^2^, Damiano Archetti^1†^, and Alberto Redolfi^1†^

# Database queries

## Pubmed query

*(depression[TIAB] OR "major depressive disorder"[TIAB] OR MDD[TIAB]) AND (multimodal[TIAB] OR "multi-modal"[TIAB]) AND ("machine learning"[TIAB] OR "deep learning"[TIAB] OR "artificial intelligence"[TIAB] OR AI[TIAB]) AND (biomarker*[TIAB] OR bioindicator*[TIAB] OR indicator*[TIAB]) AND (("neuroimaging"[TIAB] OR fMRI[TIAB] OR "functional magnetic resonance imaging"[TIAB] OR sMRI[TIAB] OR "structural MRI"[TIAB] OR DTI[TIAB] OR "diffusion tensor imaging"[TIAB] OR "brain imaging"[TIAB] OR "brain volume"[TIAB] OR "cortical thickness"[TIAB] OR "brain lesion"[TIAB]) OR (speech[TIAB] OR acoustic*[TIAB] OR voice[TIAB] OR "vocal features"[TIAB] OR prosod*[TIAB] OR "lexical feature"[TIAB] OR language[TIAB] OR text[TIAB] OR "verbal feature"[TIAB]) OR (blood[TIAB] OR biofluid*[TIAB] OR inflammatory[TIAB] OR cytokine*[TIAB] OR hormone*[TIAB] OR metabolite*[TIAB] OR genetic*[TIAB])) AND (severity[TIAB] OR "depression severity"[TIAB] OR HAMD[TIAB] OR "Hamilton Depression"[TIAB] OR BDI[TIAB] OR "Beck Depression"[TIAB] OR symptom*[TIAB] OR "clinical outcome"[TIAB] OR prediction[TIAB] OR estimation[TIAB] OR classification[TIAB])*

The previous query was integrated with another one, specific for genetics/genomics data:

*MDD AND (genetics OR genomics) AND (multimodal OR multi-modal) AND (AI OR ML OR Artificial Intelligence OR Machine Learning)*

## Web of Science query

*((TS=((depression OR "major depressive disorder" OR MDD) AND (multimodal OR multi-modal) AND ("machine learning" OR "deep learning" OR "artificial intelligence" OR AI) AND (biomarker* OR bioindicator* OR indicator*) AND (("neuroimaging" OR fMRI OR "functional magnetic resonance imaging" OR sMRI OR "structural MRI" OR DTI OR "diffusion tensor imaging" OR "brain imaging" OR "brain volume*" OR "cortical thickness" OR "brain lesion*") OR (speech OR acoustic* OR voice OR "vocal features" OR prosod* OR "lexical feature*" OR language OR text OR "verbal feature*") OR (blood OR biofluid* OR inflammatory OR cytokine* OR hormone* OR metabolite* OR genetic*)) AND (severity OR "depression severity" OR hand OR "Hamilton Depression" OR BDI OR "Beck Depression" OR symptom* OR "clinical outcome*" OR prediction OR estimation OR classification))*

*OR*

*AB=((depression OR "major depressive disorder" OR MDD) AND (multimodal OR multi-modal) AND ("machine learning" OR "deep learning" OR "artificial intelligence" OR AI) AND (biomarker* OR bioindicator* OR indicator*) AND (("neuroimaging" OR fMRI OR "functional magnetic resonance imaging" OR sMRI OR "structural MRI" OR DTI OR "diffusion tensor imaging" OR "brain imaging" OR "brain volume*" OR "cortical thickness" OR "brain lesion*") OR (speech OR acoustic* OR voice OR "vocal features" OR prosod* OR "lexical feature*" OR language OR text OR "verbal feature*") OR (blood OR biofluid* OR inflammatory OR cytokine* OR hormone* OR metabolite* OR genetic*)) AND (severity OR "depression severity" OR hand OR "Hamilton Depression" OR BDI OR "Beck Depression" OR symptom* OR "clinical outcome*" OR prediction OR estimation OR classification))*

*OR*

*TI=((depression OR "major depressive disorder" OR MDD) AND (multimodal OR multi-modal) AND ("machine learning" OR "deep learning" OR "artificial intelligence" OR AI) AND (biomarker* OR bioindicator* OR indicator*) AND (("neuroimaging" OR fMRI OR "functional magnetic resonance imaging" OR sMRI OR "structural MRI" OR DTI OR "diffusion tensor imaging" OR "brain imaging" OR "brain volume*" OR "cortical thickness" OR "brain lesion*") OR (speech OR acoustic* OR voice OR "vocal features" OR prosod* OR "lexical feature*" OR language OR text OR "verbal feature*") OR (blood OR biofluid* OR inflammatory OR cytokine* OR hormone* OR metabolite* OR genetic*)) AND (severity OR "depression severity" OR hand OR "Hamilton Depression" OR BDI OR "Beck Depression" OR symptom* OR "clinical outcome*" OR prediction OR estimation OR classification))))*

The previous query was integrated with another one, specific for genetics/genomics data:

*TS=(MDD OR "major depressive disorder") AND TS=(genetics OR genomics) AND TS=(multimodal OR "multi-modal") AND TS=(artificial intelligence OR "machine learning" OR AI OR ML)*

## Scopus query

*TITLE-ABS-KEY ((depression OR "major depressive disorder" OR MDD) AND (multimodal OR "multi-modal") AND ("machine learning" OR "deep learning" OR "artificial intelligence" OR AI) AND (biomarker* OR bioindicator* OR indicator*) AND (("neuroimaging" OR fMRI OR "functional magnetic resonance imaging" OR sMRI OR "structural MRI" OR DTI OR "diffusion tensor imaging" OR "brain imaging" OR "brain volume*" OR "cortical thickness" OR "brain lesion*") OR (speech OR acoustic* OR voice OR "vocal features" OR prosod* OR "lexical feature*" OR LANGUAGE OR text OR "verbal feature*") OR (blood OR biofluid* OR inflammatory OR cytokine* OR hormone* OR metabolite* OR genetic*)) AND (severity OR "depression severity" OR HAMD OR "Hamilton Depression" OR BDI OR "Beck Depression" OR symptom* OR "clinical outcome*" OR prediction OR estimation OR classification))*

The previous query was integrated with another one, specific for genetics/genomics data:

*TITLE-ABS-KEY(MDD OR "major depressive disorder") AND TITLE-ABS-KEY(genetics OR genomics) AND TITLE-ABS-KEY(multimodal OR "multi-modal") AND TITLE-ABS-KEY(AI OR ML OR "Artificial Intelligence" OR "Machine Learning")*

## Embase query

*('depression'/exp OR 'major depressive disorder'/exp OR MDD) AND ('multimodal'/exp OR 'multi-modal') AND ('machine learning'/exp OR 'deep learning'/exp OR 'artificial intelligence'/exp OR AI) AND (biomarker* OR bioindicator* OR indicator*) AND (('neuroimaging'/exp OR fMRI OR 'functional magnetic resonance imaging' OR sMRI OR 'structural MRI' OR DTI OR 'diffusion tensor imaging' OR 'brain imaging' OR 'brain volume*' OR 'cortical thickness' OR 'brain lesion*') OR (speech OR acoustic* OR voice OR 'vocal features' OR prosod* OR 'lexical feature*' OR language OR text OR 'verbal feature*') OR*

*(blood OR biofluid* OR inflammatory OR cytokine* OR hormone* OR metabolite* OR genetic*)) AND (severity OR 'depression severity' OR HAMD OR 'Hamilton Depression' OR BDI OR 'Beck Depression' OR symptom* OR 'clinical outcome*' OR prediction OR estimation OR classification)*

The previous query was integrated with another one, specific for genetics/genomics data:

*('major depressive disorder'/exp OR MDD) AND (genetics/exp OR genomics/exp) AND (multimodal OR multi-modal) AND (artificial intelligence/exp OR machine learning/exp OR AI OR ML)*

# Artificial Intelligence concepts explanation

- Bidirectional Long Short-Term Memory (BiLSTM): a type of neural network architecture that analyzes data sequences (like speech or text) in both forward and backward directions to better understand context and patterns.
- Constrained fusion: a data fusion approach that applies specific rules or constraints to control how different data sources are combined, ensuring the fusion process respects domain knowledge or biological/clinical relationships.
- Cross-Validation (CV): a technique for testing machine learning models by splitting data into multiple parts, training on some parts and testing on others, to ensure the model works well on new, unseen data.
- Data fusion: the process of combining information from multiple sources (such as brain scans, genetic data, and clinical assessments) to create a more complete and accurate understanding than any single source could provide alone.
- Deep Learning (DL): a subset of machine learning that uses multi-layered neural networks to automatically learn complex patterns from large amounts of data, mimicking how the human brain processes information.
- Dimensionality reduction: a technique that simplifies complex data by reducing the number of variables while preserving the most important information, making it easier to analyze and visualize without losing critical patterns.
- Ensemble voting: a method where multiple machine learning models make predictions independently, then "vote" on the final outcome, with the majority decision or averaged result typically being more accurate than any single model.
- Feature concatenation: a simple fusion method that combines features from different data sources by stacking them side-by-side into a single, longer feature vector for the model to analyze.
- Feature extraction: the process of automatically identifying and measuring relevant characteristics or patterns from raw data (like identifying specific brain regions' activity from MRI scans) that can be used for prediction or classification.
- Feature selection: the process of identifying and keeping only the most informative variables from a dataset while removing redundant or irrelevant ones, improving model performance and reducing computational complexity.
- Fine-tuning: the process of taking a pre-trained machine learning model and adjusting it slightly using new, task-specific data to adapt it for a particular application while retaining the general knowledge it learned previously.
- Large Language Models (LLMs): advanced AI systems trained on vast amounts of text data that can understand and generate human-like language.
- Leave-One-Out Cross-Validation (LOOCV): a rigorous testing method where a model is trained on all data except one sample, repeated for each sample, to thoroughly evaluate performance.
- Leave-One-Site-Out (LOSO): a validation technique for multi-center studies where data from one site is held out for testing while the model trains on all other sites, ensuring the model generalizes across different locations.
- Machine Learning (ML): a branch of AI where computers learn to make predictions or decisions from data without being explicitly programmed for every specific task.
- Natural Language Processing (NLP): AI technology that enables computers to understand, interpret, and generate human language in text or speech form.
- Neural Network (NN): a machine learning model inspired by the human brain's structure, consisting of interconnected nodes that process information to recognize patterns and make predictions.
- Random Forest (RF): a machine learning method that combines multiple decision trees to make more accurate and stable predictions, particularly effective with limited data.
- Representation-level fusion: an intermediate fusion strategy where features extracted from different data sources are combined at an abstract level within the model architecture, allowing the model to learn complex relationships between modalities.
- Self-supervised pretraining: a training approach where a model learns useful patterns from unlabeled data by solving automatically generated tasks (like predicting missing parts of an image), before being applied to a specific task with limited labeled data.
- Self-supervised training: a learning method where the model creates its own training labels from the data structure itself, enabling it to learn from large amounts of data without requiring human annotation.
- SHapley Additive exPlanations (SHAP): a technique for explaining machine learning model predictions by showing which features (input variables) contributed most to each individual decision.
- Support Vector Machines (SVM): a machine learning algorithm that finds the optimal boundary to separate different groups in data, commonly used for classification tasks in medical diagnosis.
